# Alleviation of 4-nitroquinoline 1-oxide induced oxidative stress by *Oroxylum indicum* (L.) leaf extract in albino Wistar rats

**DOI:** 10.1186/s12906-016-1186-x

**Published:** 2016-07-19

**Authors:** Shalini Mohan, Kalaivani Thiagarajan, Balaji Sundaramoorthy, Vivek Gurung, Manas Barpande, Shilpi Agarwal, Rajasekaran Chandrasekaran

**Affiliations:** Department of Plant Biotechnology, School of Bio Sciences and Technology, VIT University, Vellore, 632014 Tamil Nadu India

**Keywords:** *Oroxylum indicum* (L.), 4-nitroquinoline 1-oxide, Oxidative stress, Anticancer, Antioxidant

## Abstract

**Background:**

4-nitroquinoline 1-oxide (4-NQO) is a mutagen known to be responsible for causing cancer by generating oxidative stress in humans. *Oroxylum indicum* (L.) possesses various bioactive compounds with antioxidant properties. In this connection, the present study aims to analyze the alleviation of 4-NQO induced oxidative stress in albino Wistar rats using *O. indicum* (L.) leaf extract.

**Methods:**

*O. indicum* (L.) belonging to the family Bignoniaceae, has anticancer and anti-inflammatory properties. In this study, we observed severe oxidative stress in 4-NQO induced albino Wistar rats when compared to untreated control. Alleviation of this condition was seen after the oral administration of *O. indicum* (L.) leaf extract at 50, 100, and 200 mg/kg body weight.

**Results:**

4-NQO (50 ppm) administration in drinking water resulted in the generation of reactive oxygen species (ROS) leading to cellular damage, lipid peroxidation and imbalance in antioxidant status. Administration of *O. indicum* (L.) leaf extract has alleviated the level of 4-NQO induced oxidative stress by increasing the antioxidant status and decreasing the elevation of liver markers in serum.

**Conclusions:**

Results clearly suggest that *O. indicum* (L.) leaf extract when administered orally in a dose dependent manner has the ability to overcome the oxidative stress induced by 4-NQO with hepatoprotective and lipid protective properties.

## Background

4-nitroquinoline 1-oxide (4-NQO) is considered as a potent carcinogen and a good experimental model for studying oral carcinogenesis [1]. 4-NQO causes single strand breaks (SSB) in vivo leading to oxidative damage, through the formation of ROS [2]. It also depletes the glutathione level in cells. Oxidative stress accounts for the disturbance in the balance between ROS and antioxidants. ROS are highly reactive molecules that contain oxygen free radicals such as superoxide radicals, hydroxyl radicals and hydrogen peroxide (H_2_O_2_). Increase in ROS causes cellular damage, damage to DNA, proteins and fats [3]. It also leads to lipid peroxidation by attacking membrane lipids [4]. This further leads to the aging process and degenerative diseases such as cardiovascular diseases, immune dysfunction, Alzheimer’s disease, diabetes, and cancer [5].

The removal of H_2_O_2_ or other hydroperoxides by glutathione peroxidase (GPx) requires reduced glutathione (GSH) as cofactor. GPx converts H_2_O_2_ to H_2_O and GSH to oxidized glutathione (GSSG) simultaneously. GSSG is restored to a reduced form by glutathione reductase (GR). This reaction serves to maintain a high GSH/GSSG ratio in the cell. It leads to detoxification of the lipid peroxidation effects. SOD converts superoxide radical into peroxides, whereas GPx and catalase (CAT) convert peroxide into water. In general, the oral cavity is susceptible to free radical damage due to rapid absorption in the mucous membrane contributing to oxidative stress and inflammation. In turn, antioxidants nullify the damage by donating an electron which stabilizes the vacancy in the outermost shell of ROS. Glutathione, vitamin C, vitamin E, vitamin A and various enzymes such as CAT, SOD, and GPx are natural antioxidants. Synthetic antioxidants are toxic and therefore natural antioxidants are more preferred [6]. Flower buds of *Piper cubeba* (L.) showed anti-free radical damage through SOD and CAT [7]. Various fractions of 95 % methanol extract of *Torilis leptophylla* (L.) showed antioxidant activity and cytoprotectivity due to free radical scavenging property [8].

*Oroxylum indicum* (L.) is a tropical deciduous tree of 12 meters height belongs to the family Bignoniaceae and is found in India, Sri Lanka, Japan, China, Malaysia and Bhutan [9]. In India, this plant is mainly found in North-East, Himalayan foothills and the Western and Eastern Ghats. Various parts of this plant is used to treat various diseases like diarrhea, fever, cancer, ulcer and jaundice. This plant is also used as analgesic, anti-inflammatory and antitussive agent [10]. *O. indicum* (L.) has also been reported to significantly alleviate the symptoms of colitis in experimental rats [11]. *O. indicum* (L.) is a rich source of chrysin, oroxylin-A, scutellarin, and baicalein which are medicinally important bioactive compounds [12]. Chrysin and baicalein have antibacterial [3, 13] and anticancer properties [14–16].

Methanol extract of *O. indicum* (L.) has shown anti-proliferative properties by increasing the expression of p53 thus enhancing apoptosis [17]. Ethanol extracts of *O. indicum* (L.) fruit and stem bark containchrysin, oroxylin-A and baicalien, oroxyloside methyl ester and chrysin-7-O- methyl glucoside which are known to possess anti-malignant properties. Also, the polar bark extracts of *O. indicum* (L.) possess antiproliferative and cytotoxic properties against the human breast cancer cells. Few other studies have also shown that non-polar extract of *O. indicum* (L.) possesses apoptosis promoting ability due to few bioactive phytochemicals present in the extract [18]. In the present study, the effect of *O. indicum* (L.) leaf extract was evaluated against 4-NQO induced oxidative stress in a dose-dependent manner against normal control using albino Wistar rats.

## Methods

### Plant material

Leaves of *O. indicum* (L.) were collected from Kottayam region, Kerala, India. *O. indium* (L.) was identified by Dr G.V.S. Murthy and a voucher specimen number 1036 was deposited at Botanical Survey of India, Southern Regional Centre, TNAU campus, Coimbatore.

### Preparation of *O. indicum* (L.) leaf extract

*O. indicum* (L.) leaves were cleaned and air dried at room temperature. 25 g of *O. indicum* (L.) leaf powder was placed in the Soxhlet apparatus for extraction using 150 ml of 99.9 % ethanol in the ratio of 1:6. The extract was filtered and concentrated using rotary evaporator and refrigerated till use [19]. 25 g of powder yielded around 1.5 g of ethanol extract (6 %). These results are in accordance with the previous report on extraction efficiency of different solvents from *O. indicum* (L.) leaves [20, 21].

### Experimental animals

Adult male Wistar rats were used for the study. All experiments were approved by the Institutional Animal Ethics Committee (IAEC), Vellore Institute of Technology (VIT) University, Tamil Nadu, India (VIT/IAEC/VII^th^/23). The animals were maintained at 23 °C on a 12 h light/dark cycle with free access to water and food. 4-NQO was purchased from Sigma Aldrich (St Louis, MO). 5, 5′-dithiobis-(2- nitrobenzoic acid) (DTNB), glutathione reduced (GSH) were purchased from Himedia, Mumbai, India. Diagnostic kits for liver markers, cholesterol and proteins were purchased from Autospan diagnostic kits. All other chemicals used were of analytical grade.

### Study design

Thirty rats of 6–8 weeks old were grouped into 5 containing 6 animals each.

**Table:** 

Group 1	Oral administration of 1 ml of 0.1 % ethanol (normal control)
Group 2	Administration of 50 ppm 4-NQO in drinking water for 4 months
Group 3	50 mg/kg body weight (bw) of *O. indicum* (L.) leaf extract for a month after 4-NQO administration
Group 4	100 mg/kg bw of *O. indicum* (L.) leaf extract for a month after 4-NQO administration
Group 5	200 mg/kg bw of *O. indicum* (L.) leaf extract for a month after 4-NQO administration

At the end of the experiment, all the animals were sacrificed, blood was collected, centrifuged and the serum was exposed to various biochemical assays. Liver was also dissected and homogenized for lipid peroxidation and antioxidant analyses.

### Biochemical analysis

#### Evaluation of lipid peroxidation (LPO) in liver

LPO levels were measured as thiobarbituric reactive acid substances (TBARS) according to the method described by Ohkawa and collaborators (1979) [22]. The absorbance of pink color formation in the supernatant was measured at 535 nm.

### Antioxidant status in liver

SOD activity, which is based on the inhibition of reduction of nitro blue tetrazolium (NBT) to blue formazan was measured according to Kakkar et al., (1984) [23] with modification. The absorbance of formazan in the supernatant was measured at 519 nm. The CAT activity in liver homogenate was carried out according to Sinha 1972 [24]. The absorbance was measured at 620 nm. Determination of GPx level in liver was carried out according to Rotruck (1973) [25]. Reduced glutathione levels were assayed according to Ellman et al., (1961) [26]. The absorbance of GPx and GSH was measured at 650 nm. Vitamin C was measured according to Omaye et al., (1979) [27]. The absorbance was read at 530 mm. Vitamin E was assayed according to Baker and Frank (1988) [28]. The absorbance was read at 520 nm.

### Assessment of serum markers

Total protein, albumin, glucose, creatinine, liver markers such as alanine transaminase (ALT), alkaline phosphatase (ALP), aspartate transaminase (AST) and total bilirubin followed by total cholesterol, high density lipoprotein (HDL) and triglycerides were measured using Span diagnostic reagent kits [29].

### Statistical analysis

Statistical differences among groups were analyzed by one-way analysis of variance (ANOVA) followed by Duncan’s multiple range test (DMRT). Groups were considered statistically significant when *p* < 0.05 [29].

## Results

### Evaluation of lipid peroxidation (LPO) in liver

**Fig. 1:**
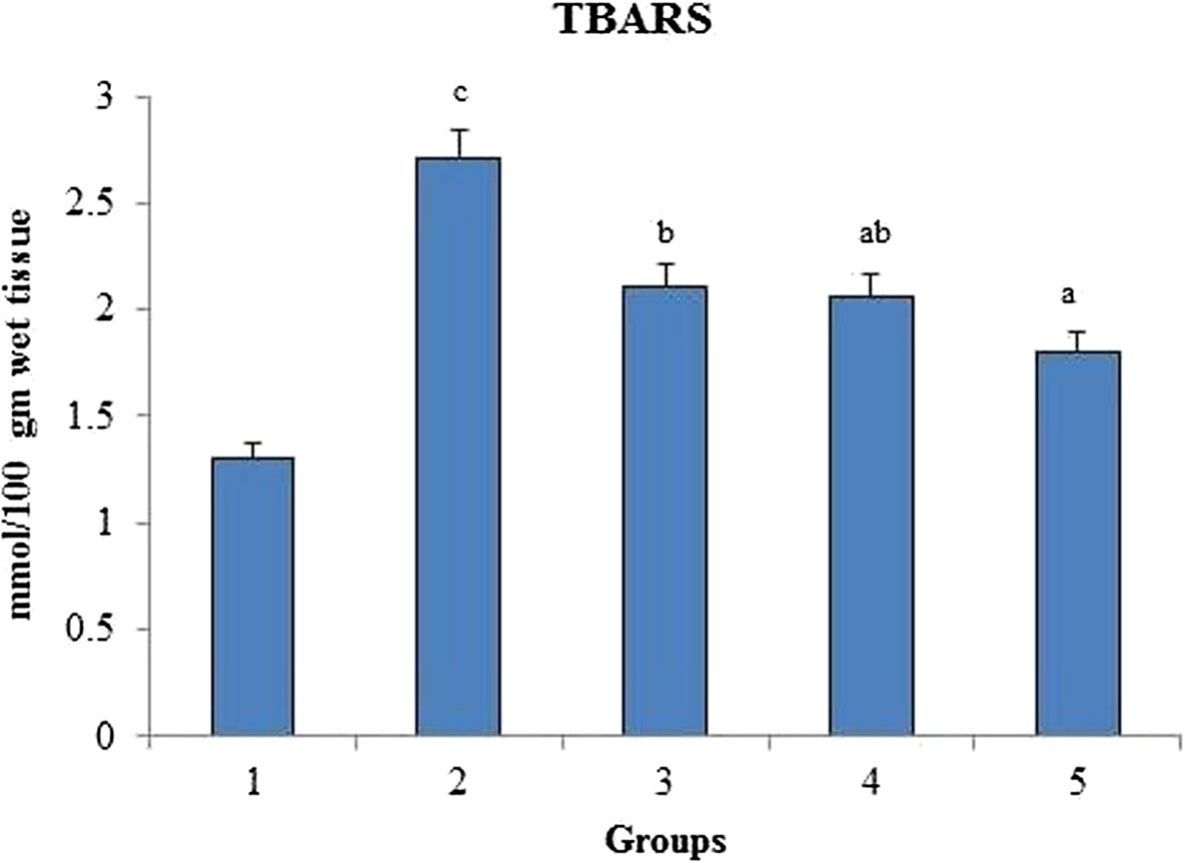
Effect of *O. indicum* (L.) leaf extract on lipid peroxidation against 4-NQO induced oxidative stress. Each value represents mean ± SD in each group. Bars not sharing a common letter (a-c) differ significantly with normal control and with each other (*p* < 0.05, Duncan’s multiple range test (DMRT)). Group 1 - Control, Group 2- 4-NQO (50 ppm), Groups 3–5 - *O. indicum* (L.) leaf extract – 50, 100 and 200 mg/kg body weight

Figure 1 shows the effect of *O. indicum* (L.) leaf extract on TBARS level in liver of 4-NQO induced oxidative stress in rats. The results show a significant increase in TBARS level in the liver homogenate of group 2. However, the increase in TBARS level was significantly reduced in the liver of rats orally administered with *O. indicum* (L.) leaf extract. A significant difference was also seen between group 5 and group 1 control.

### Antioxidant status in liver

**Fig. 2:**
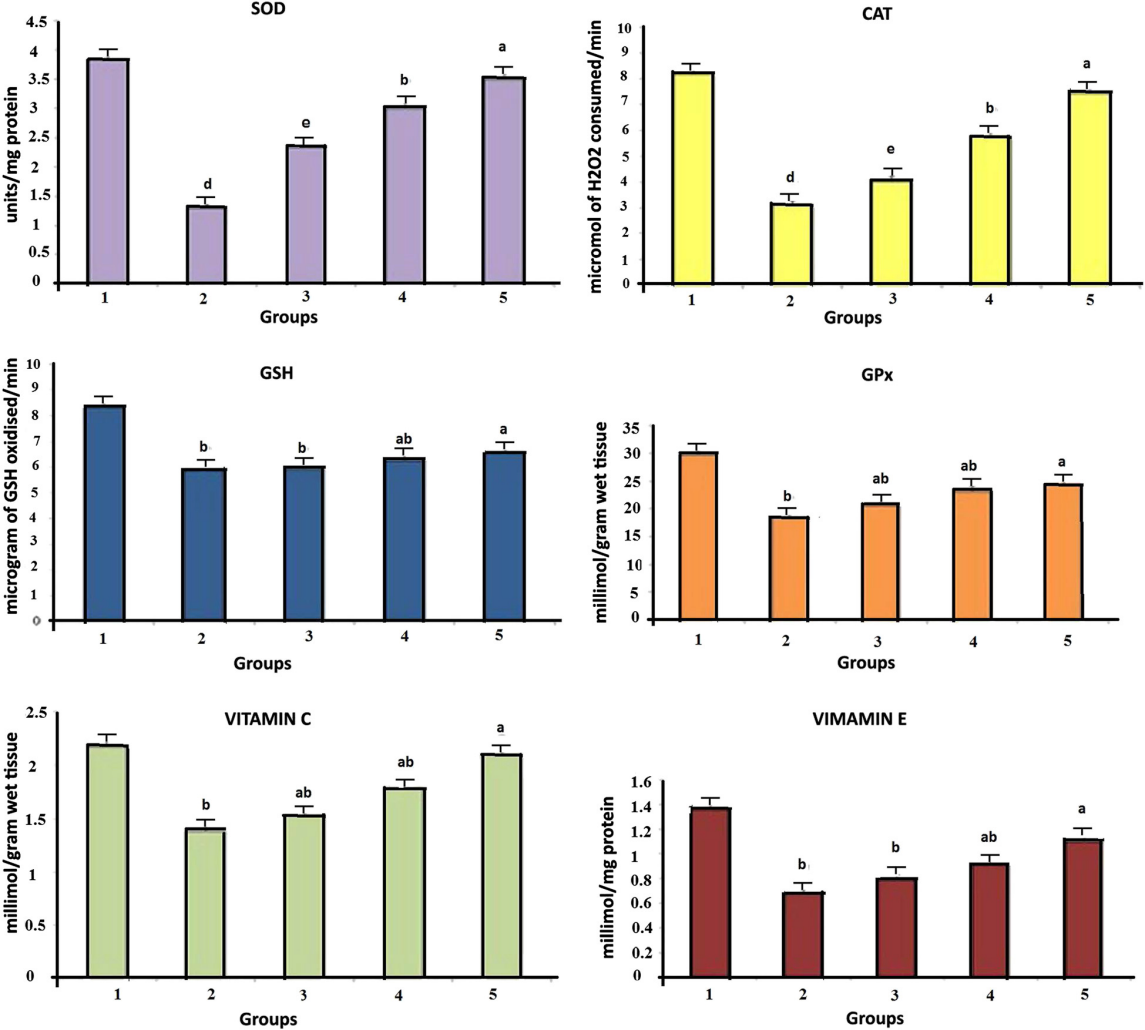
Effect of *O. indicum* (L.) leaf extract on antioxidants against 4-NQO induced oxidative stress. Each value represents mean ± SD in each group. Bars not sharing a common letter (a-d) differ significantly with normal control and with each other (*p* < 0.05, Duncan’s multiple range test (DMRT)). Group 1 - Control, Group 2- 4-NQO (50 ppm), Groups 3–5 - *O. indicum* (L.) leaf extract – 50, 100 and 200 mg/kg body weight

Figure 2 shows the effect of *O. indicum* (L.) leaf extract on antioxidant levels (SOD, CAT, GPx, GSH, vitamin C and vitamin E) against 4-NQO induced oxidative stress in the liver of albino Wistar rats. Results revealed a highly significant difference between group 1 and group 2. Groups treated with *O. indicum* (L.) leaf extract showed a descending order of significant differences from groups 3–5 when compared to group 1control.

### Assessment of serum liver markers

**Fig. 3:**
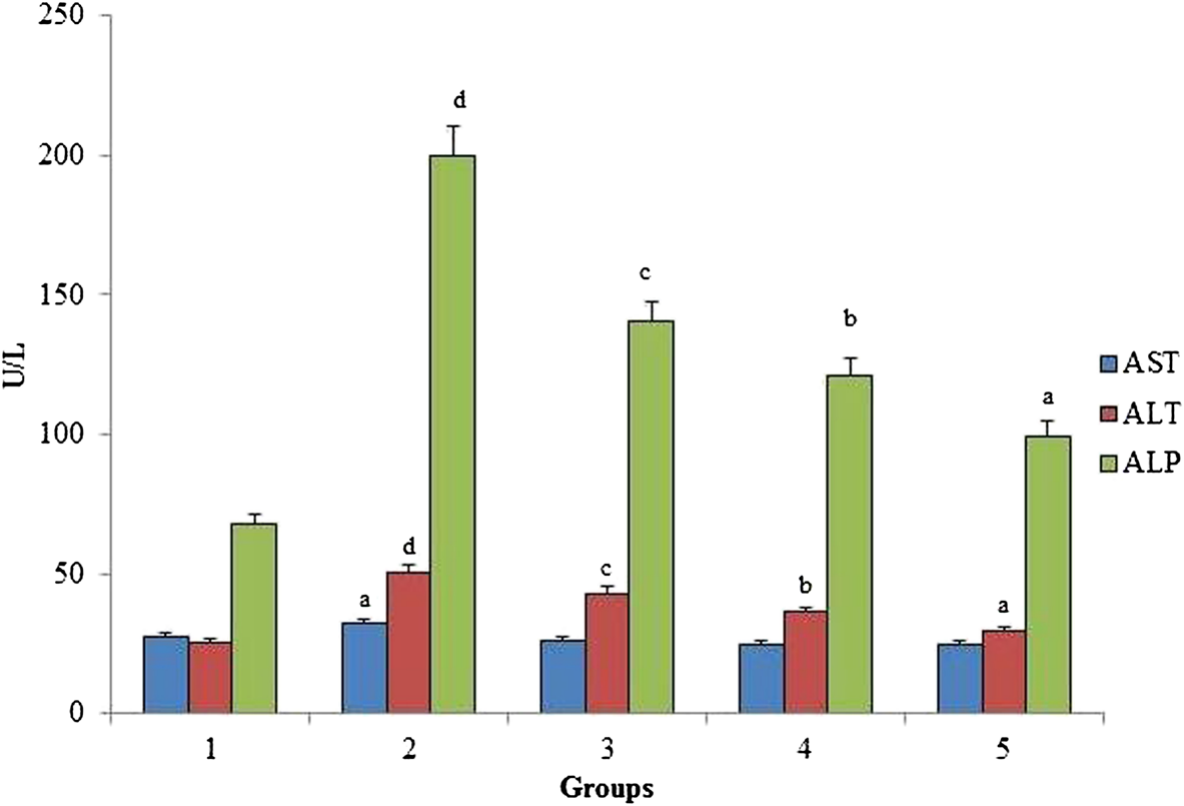
Effect of *O. indicum* (L.) leaf extract on liver markers against 4-NQO induced oxidative stress. Each value represents mean ± SD in each group. Bars not sharing a common letter (a-d) differ significantly with normal control and with each other (*p* < 0.05, Duncan’s multiple range test (DMRT)). Group 1 - Control, Group 2- 4-NQO (50 ppm), Groups 3–5 - *O. indicum* (L.) leaf extract – 50, 100 and 200 mg/kg body weight

**Fig. 4:**
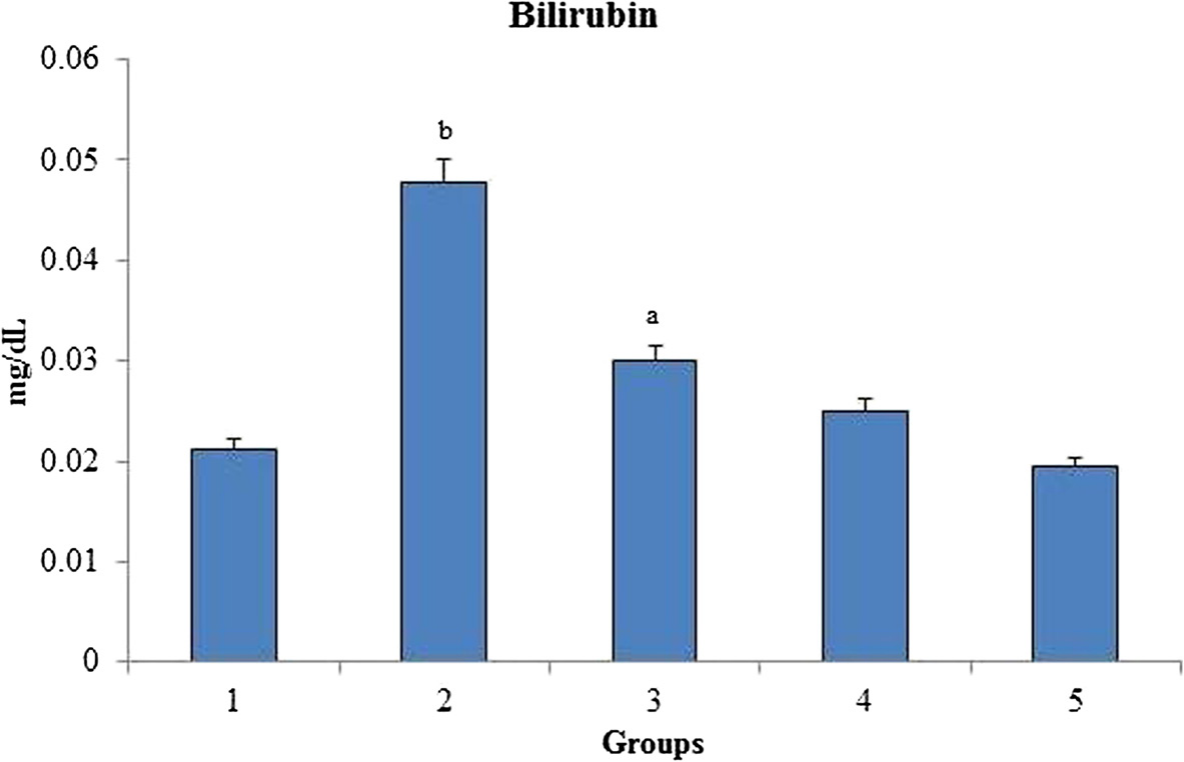
Effect of *O. indicum* (L.) leaf extract on total bilirubin against 4-NQO induced oxidative stress. Each value represents mean ± SD in each group. Bars not sharing a common letter (a and b) differ significantly with normal control and with each other (*p* < 0.05, Duncan’s multiple range test (DMRT)). Group 1 - Control, Group 2- 4-NQO (50 ppm), Groups 3–5 - *O. indicum* (L.) leaf extract – 50, 100 and 200 mg/kg body weight

Figure 3 shows the effect of *O. indicum* (L.) leaf extract on serum liver markers (AST, ALT, ALP and total bilirubin) against 4-NQO induced oxidative stress in rats. Results showed a significant increase in the level of liver markers in group 2. The level of liver markers were decreased significantly upon the oral administration of *O. indicum* (L.) leaf extract. Figure 4 shows a significant increase in the bilirubin level of group 2 when compared to group 1 reflecting the induction of hyperbilirubinemia. The level of bilirubin was found to decrease to near normal level in constant proportions with group 5 having bilirubin level nearest to the control group 1.

### Creatinine

**Fig. 5:**
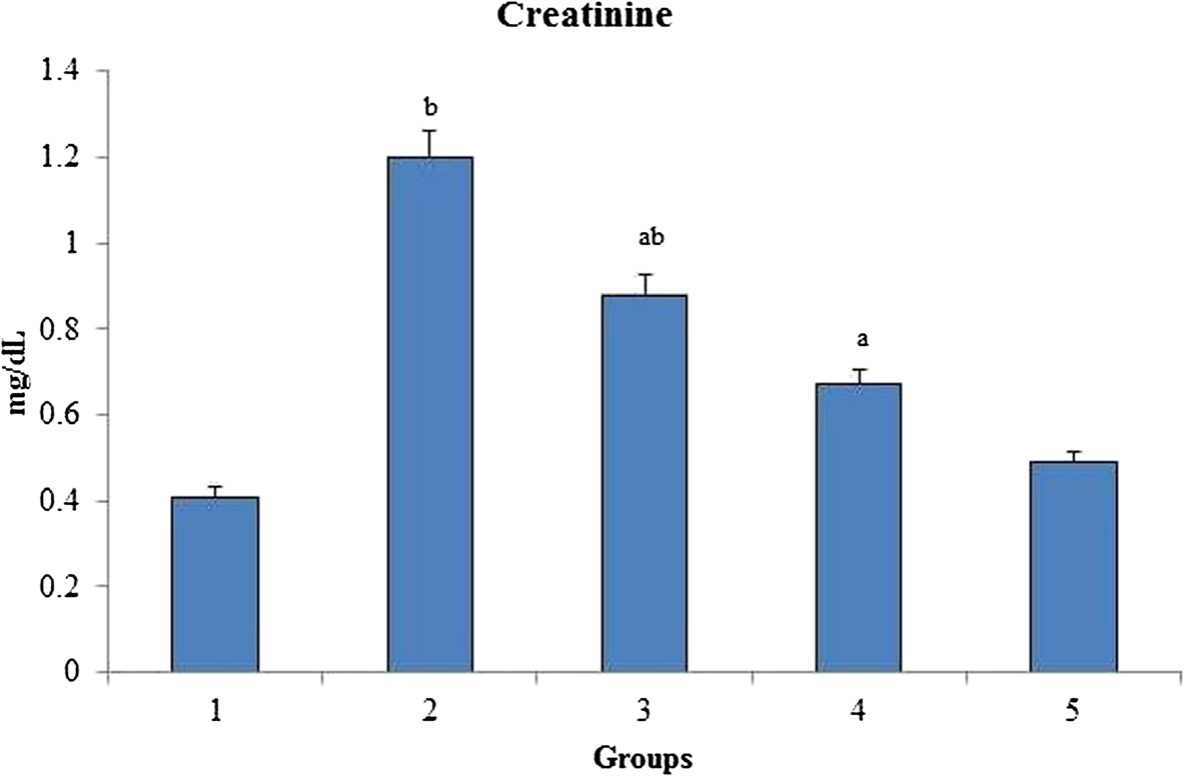
Effect of *O. indicum* (L.) leaf extract on creatinine against 4-NQO induced oxidative stress. Each value represents mean ± SD in each group. Bars not sharing a common letter (a and b) differ significantly with normal control and with each other (*p* < 0.05, Duncan’s multiple range test (DMRT)). Group 1 - Control, Group 2- 4-NQO (50 ppm), Groups 3–5 - *O. indicum* (L.) leaf extract – 50, 100 and 200 mg/kg body weight

There was a significant increase in the creatinine level of group 2 when compared to group 1 (Fig. 5). When the rats were orally administered with *O. indicum* (L.) leaf extract, the levels of creatinine were found to decrease to near normal. The creatinine level was seen to be increased by almost two fold when compared to the normal control.

### Total protein and albumin

**Fig. 6:**
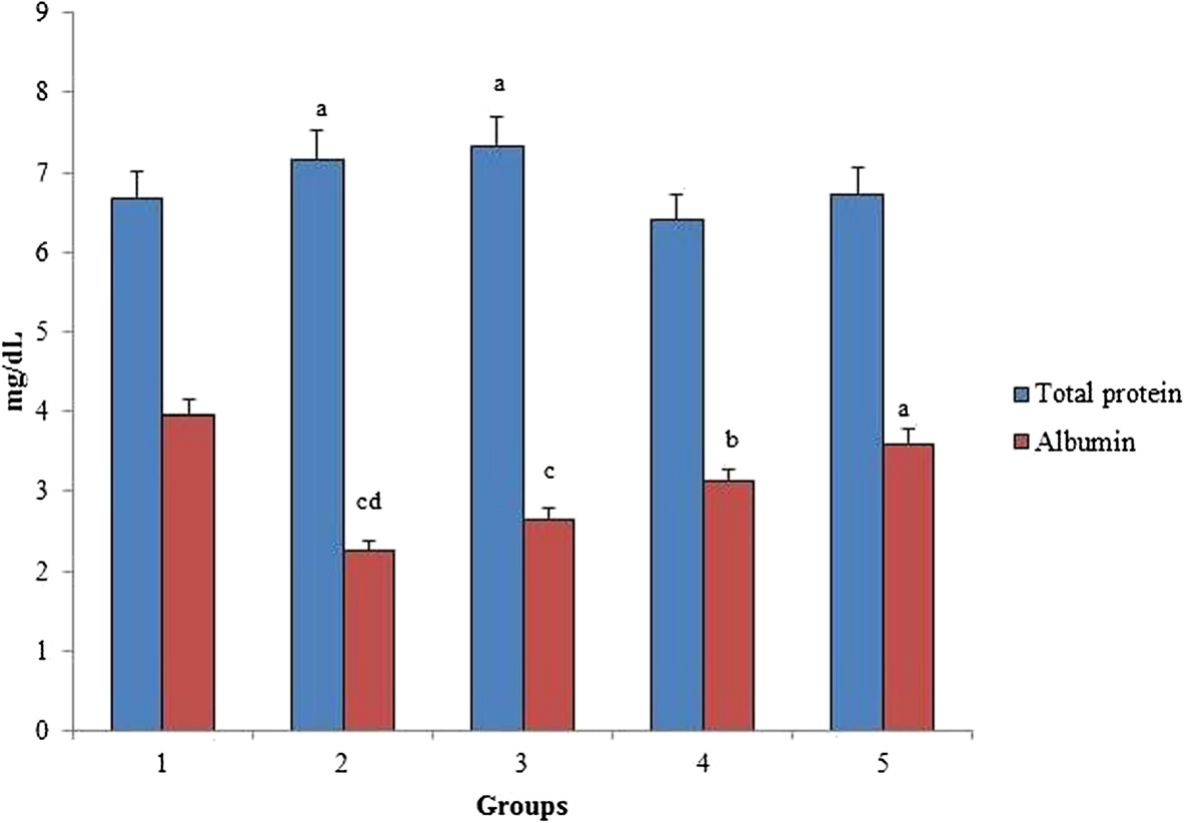
Effect of *O. indicum* (L.) leaf extract on total serum proteins against 4-NQO induced oxidative stress. Each value represents mean ± SD in each group. Bars not sharing a common letter (a-d) differ significantly with normal control and with each other (*p* < 0.05, Duncan’s multiple range test (DMRT)). Group 1 - Control, Group 2- 4-NQO (50 ppm), Groups 3–5 - *O. indicum* (L.) leaf extract – 50, 100 and 200 mg/kg body weight

Figure 6 shows a reduction in total protein and albumin levels in group 2 due to the administration of 4-NQO. When rats were treated with *O. indicum* (L.) leaf extract, the levels were significantly increased in groups 3–5.

### Glucose

**Fig. 7:**
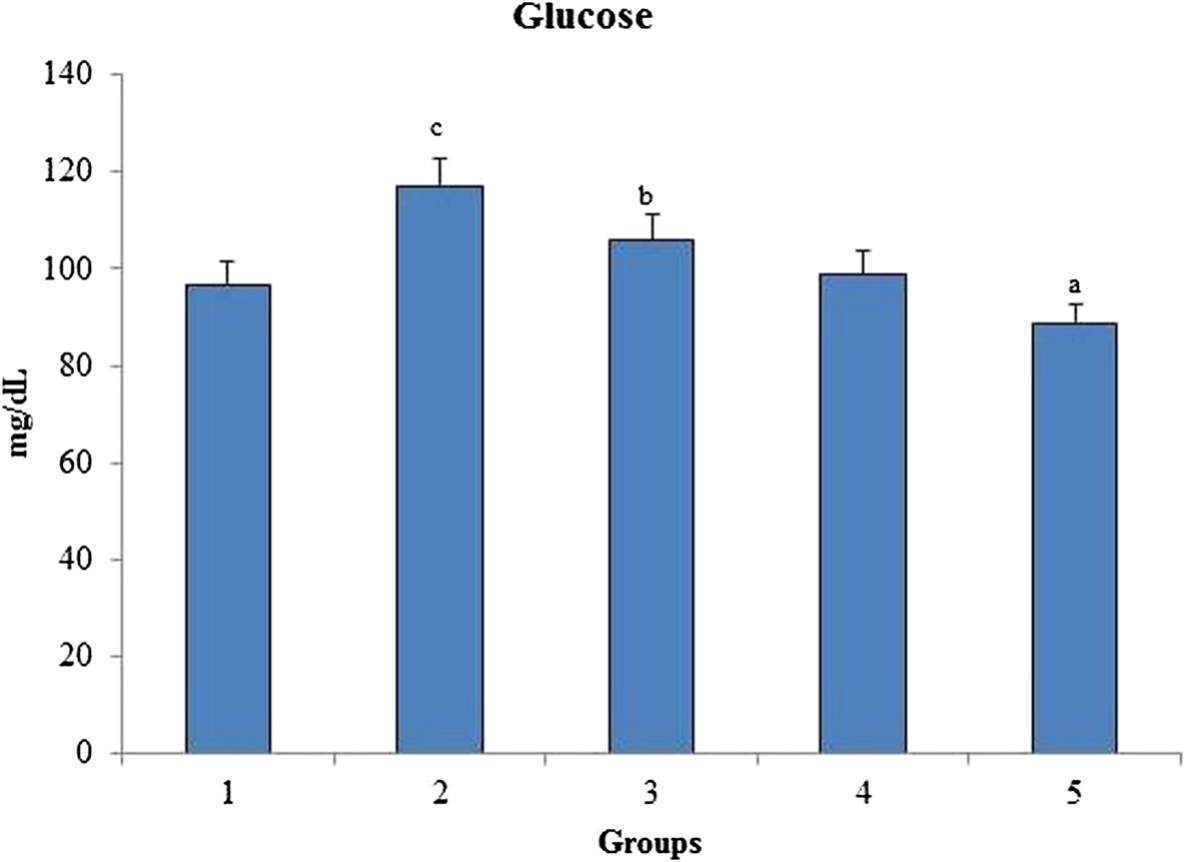
Effect of *O. indicum* (L.) leaf extract on serum glucose against 4-NQO induced oxidative stress. Each value represents mean ± SD in each group. Bars not sharing a common letter (a-c) differ significantly with normal control and with each other (*p* < 0.05, Duncan’s multiple range test (DMRT)). Group 1 - Control, Group 2- 4-NQO (50 ppm), Groups 3–5 - *O. indicum* (L.) leaf extract – 50, 100 and 200 mg/kg body weight

Glucose level increases linearly with increase in oxidative stress burden. A significant increase in glucose level of group 2 was seen when compared to group 1 control reflecting the induction of hyperglycaemia. When the rats were orally administered with *O. indicum* (L.) leaf extract, the levels of glucose was brought back to near normal level in constant proportions with group 5 having glucose level nearest to group 1 control (Fig. 7).

### Total cholesterol

**Fig. 8:**
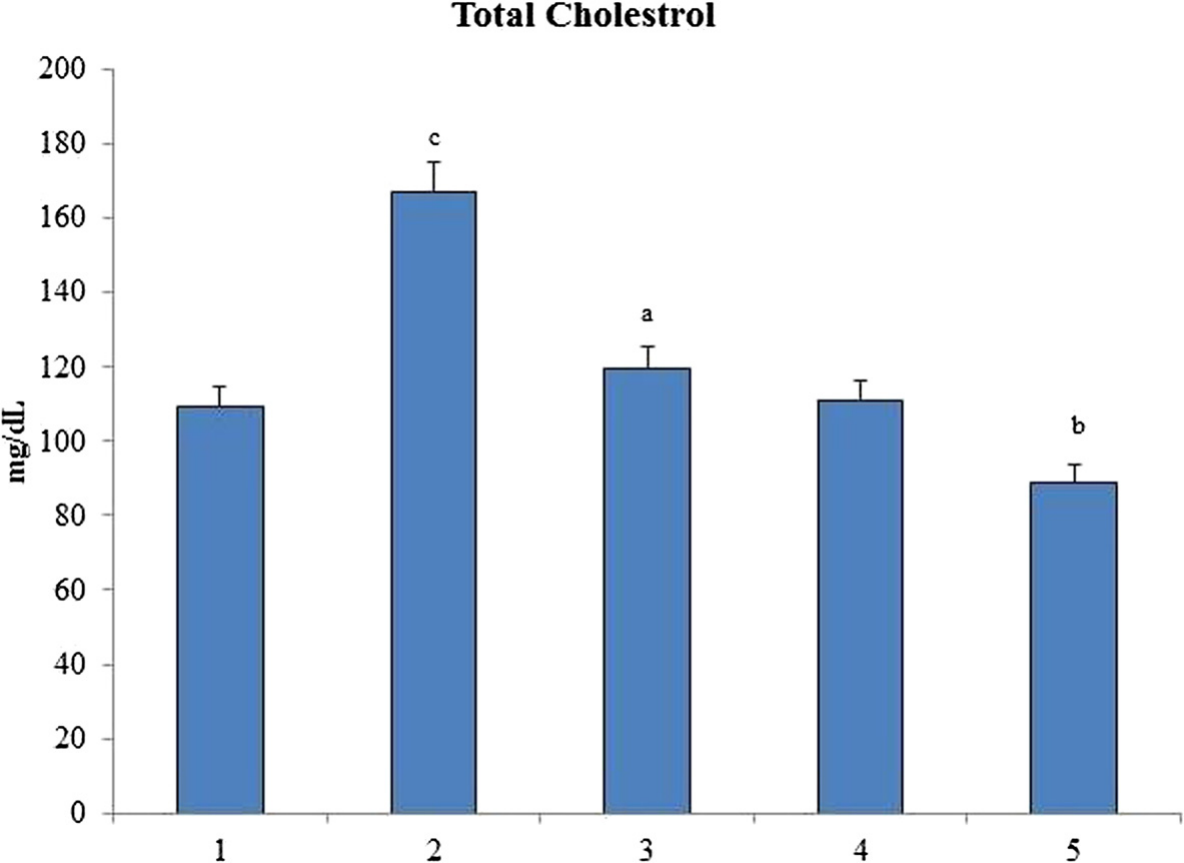
Effect of *O. indicum* (L.) leaf extract on total cholesterol against 4-NQO induced oxidative stress. Each value represents mean ± SD in each group. Bars not sharing a common letter (a-c) differ significantly with normal control and with each other (*p* < 0.05, Duncan’s multiple range test (DMRT)). Group 1 - Control, Group 2- 4-NQO (50 ppm), Groups 3–5 - *O. indicum* (L.) leaf extract – 50, 100 and 200 mg/kg body weight

Total cholesterol level increases linearly with increase in oxidative stress burden. A significant increase in the total cholesterol level of group 2 was noticed when compared to group 1 control reflecting the induction of hypercholesterolemia. When the rats were orally administered with *O. indicum* (L.) leaf extract the level of total cholesterol was found to decrease to near normal level in constant proportions with group 4 having cholesterol level nearest to the control group 1 (Fig. 8).

### High density lipoprotein (HDL)

**Fig. 9:**
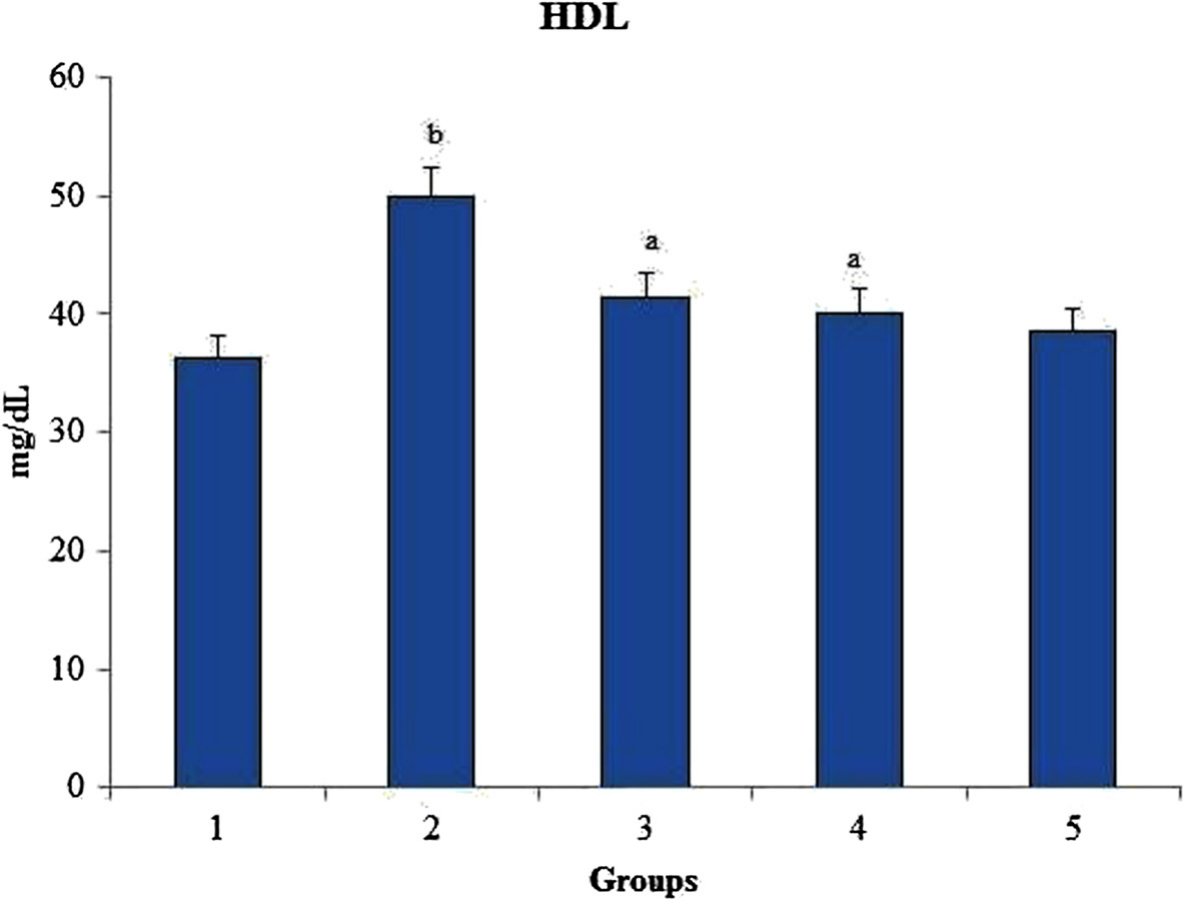
Effect of *O. indicum* (L.) leaf extract on high density lipoprotein against 4-NQO induced oxidative stress. Each value represents mean ± SD in each group. Bars not sharing a common letter (a and b) differ significantly with normal control and with each other (*p* < 0.05, Duncan’s multiple range test (DMRT)). Group 1 - Control, Group 2- 4-NQO (50 ppm), Groups 3–5 - *O. indicum* (L.) leaf extract – 50, 100 and 200 mg/kg body weight

HDL levels were found to increase linearly with increase in oxidative stress burden. There was a significant increase in the HDL level of group 2 when compared to group 1 reflecting the induction of hyperalphalipoproteinemia. When the rats were orally administered with *O. indicum* (L.) leaf extract the levels of HDL levels were found to decrease to near normal as seen in Fig. 9.

### Triglycerides

**Fig. 10:**
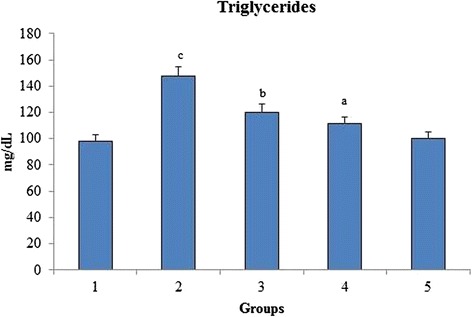
Effect of *O. indicum* (L.) leaf extract on triglycerides against 4-NQO induced oxidative stress. Each value represents mean ± SD in each group. Bars not sharing a common letter (a-c) differ significantly with normal control and with each other (*p* < 0.05, Duncan’s multiple range test (DMRT)). Group 1 - Control, Group 2- 4-NQO (50 ppm), Groups 3–5 - *O. indicum* (L.) leaf extract – 50, 100 and 200 mg/kg body weight

A significant increase in triglycerides level of group 2 was seen when compared to group 1 reflecting the induction of hypertriglyceridaemia (Fig. 10). When the rats were orally administered with *O. indicum* (L.) leaf extract the levels of triglycerides were found to decrease to near normal in constant proportions with group 5 having triglycerides level nearest to the control group 1.

## Discussion

4-NQO is known as a potent carcinogen and exhibits oxidative stress inducing property by generating superoxide and hydrogen peroxide as free radicals [30]. It reacts with DNA to form stable quinoline mono adducts and thus results in mutagenicity and genotoxicity. This study mainly focuses on the protective effect of *O. indicum* (L.) leaf extract against oxidative stress generated by 4-NQO in rats.

The present study was conducted based on the previous report on *O. indicum* (L.) for antioxidant and hepatoprotectivity using *in vitro* models [21]. Literature review suggests that *O. indicum* (L.) have not shown any toxicity to humans and experimental animals at higher doses [31]. Based on the previous acute oral toxicity study conducted according to the Organisation for Economic Co-operation and Development (OECD) guidelines, 300 mg/kg of *O. indicum* (L.) ethanolic leaf extract was hepatoprotective and non-toxic [21]. On this basis, doses lesser than 300 mg/kg such as 50, 100 and 200 mg/kg were chosen for the present study.

From the results, the level of TBARS was significantly increased in the liver of group 2 rats induced with 4-NQO when compared to group 1 normal control. There is a direct relation between lipid peroxidation and TBARS [32]. This increase in stress was found to be decreased in groups 3–5 indicating the protection offered by *O. indicum* (L.) leaf extract to lipid membrane in spite of the dose administered.

The enzymes SOD, CAT, GSH and GPx are at particularly high levels in liver and serve in detoxification mechanism. The level of these enzymes was found to be decreased in liver tissues of group 2 when compared to group 1 normal control due to the oxidative stress caused by 4-NQO. The level of antioxidants was found to be increased with increasing concentration of *O. indicum* (L.) leaf extract in groups 3–5, thus inferring the antioxidant effect of *O. indicum* (L.) leaf extract. In general, polyphenols present in plant extracts possess many hydroxyl groups including O-dihydroxy group which have very strong radical scavenging effect and antioxidant power [33]. *O. indicum* (L.) has been chosen due to the presence of various bioactive compounds [34]. *O. indicum* (L.) has high amount of flavones (oroxylin A, chrysin, baicalein, baicalin) which are known for their high antioxidant property [35].

Oxidative damage in DNA can cause cancer and vitamin C and vitamin E play an important and valuable role in preventing the disease [36]. The level of vitamin C and E was found in a similar fashion as other antioxidant enzymes. Low levels of vitamin C and E was observed in group 2 thus showing deleterious effect of 4-NQO. On the other hand, in groups 3–5, these levels were found to be increased in constant proportion thereby confirming the protective activity of the *O. indicum* (L.) leaf extract.

Biochemical changes in the blood are important indicators of the toxicity profile of important compounds [37]. The presence of transaminases (AST, ALT and ALP) in the cytosol or mitochondria indicates serious hepatocellular damage or changes in the membrane permeability [38]. The hazardous effects of 4-NQO on liver are well known and seem to be directly related to increase the concentration of hepatic enzymes that in turn, lead to hepatic necrosis. Earlier studies have also shown that 4-NQO induced rats significantly increase the level of hepatic markers (ALP, AST and ALT) in serum because it causes damage to hepatocytes leading to the outflow of enzymes [39]. The present study shows a similar effect of 4-NQO on rats i.e., the level of hepatic markers was found to be increased in group 2 and the levels were decreased after the administration of *O. indicum* (L.) leaf extract in groups 3–5. It can be seen that *O. indicum* (L.) leaf extract causes reduction in the activity of enzymes induced by 4-NQO thus appearing as an effective anti-hepatotoxic agent and protecting liver from further damage. Similar results of *O. indicum* (L.) leaf extract was reported by Harminder et al., [40] who studied that root bark of the plant has hepatoprotective activity against carbon tetrachloride induced lipid degradation in mice and rats. Also, Deka et al., [34] had stated in his paper that ethanolic extract of leaves and fruits of *O. indicum* (L.) exhibited anti-hepatotoxic property. The serum bilirubin level was found to be increased in group 2 when compared to the normal control, but it was still within the normal range (0.2-0.55 mg/dL). The bilirubin level was found to be low in constant proportions with increasing *O. indicum* (L.) leaf extract concentrations [41]. As seen in Fig. 5, significant difference was observed in the concentration of creatinine in the 4-NQO induced groups as compared to the group 1 control, thereby proving that 4-NQO induces oxidative stress leading to kidney impairment.

Based on the previous report, the in vitro antioxidant and hepatoprotective activities were due to the presence of polar phenolic flavonoid compounds and tannins etc. Also *O. indicum* (L.) was reported to possess high amount of flavones such as oroxylin A, chrysin, baicalein, baicalin etc. which are known for their high antioxidant property [34, 35]. Among these compounds, baicalin is reported to protect the liver from CCl_4_-induced oxidative stress and can be consumed for long time period without any known side effects and a promising therapeutic in human oxidative stress [42].

In addition to the above experiments, the levels of total protein and albumin were found to be significantly different in group 2 when compared to group 1 control. As depicted in Fig. 6, the increase in total serum proteins was directly proportional to the increase in the concentration of *O. indicum* (L.) leaf extract. Figure 7 shows the serum glucose level in all the groups. The serum glucose level was found to be very high in group 2 when compared to group 1 control, but it was still found to be in the normal glucose range (50–135 mg/dL). No significant difference in glucose levels was observed in all the treated groups when compared with the inducer and normal.

Total cholesterol and HDL levels increased linearly with the oxidative stress burden. Group 2 rats showed abnormally high levels of total cholesterol and HDL bordering on the verge of hypercholesterolemia and hyperalphalipoproteinemia respectively. Both the total cholesterol and HDL levels were found to be low with increasing concentrations of *O. indicum* (L.) leaf extract in a constant proportion. A significant increase in the triglycerides level was observed in group 2 when compared to group 1, reflecting the induction of hypertriglyceridaemia. *O. indicum* (L.) leaf extract successfully lowered the serum triglycerides level with increasing concentration of *O. indicum* (L.) leaf extract in groups 3–5 to almost similar to the control group 1. The mechanism by which the triglyceride level was lowered by *O. indicum* (L.) leaf extract is still unknown. It can be hypothesized that this was achieved by increasing the fatty acid oxidation [43].

## Conclusions

The biochemical analysis done in the present study is an evidence for 4-NQO induced oxidative stress leading to structural and functional damage to tissues. *O. indicum* (L.) leaf extract used in this research work successfully inhibited the action of the inducer 4-NQO and showed anti-hepatotoxic, antioxidant and renal protective activity. Thus, the overall study suggests a dose dependent protection by *O. indicum* (L.) leaf extract against 4-NQO induced oxidative stress in rats. Considering the earlier reports on bioactivity of different compounds present in *O. indicum* (L.), we emphasize that, baicalin may be the potential candidate for hepatoprotection against oxidative stress and therefore needs further validation.

## Abbreviations

4-NQO, 4-nitroquinoline 1-oxide; ALP, alkaline phosphatase; ALT, alanine transaminase; AST, aspartate transaminase; CAT, catalase; DMRT, Duncan’s multiple range test; DTNB, 5, 5′-dithiobis-(2- nitrobenzoic acid); GPx, glutathione peroxidase; GR, glutathione reductase; GSH, reduced glutathione; GSSG, oxidized glutathione; H_2_O_2_, hydrogen peroxide; HDL, high density lipoprotein; IAEC, Institutional Animal Ethics Committee; NBT, nitro blue tetrazolium; OECD, Organisation for Economic Co-operation and Development; ROS, reactive oxygen species; SOD, superoxide dismutase; SSB, single strand breaks; TBARS, thiobarbituric acid reactive substances; TNAU, Tamil Nadu Agriculture University.
