# Correction to: Alleviation of 4-nitroquinoline 1-oxide induced oxidative stress by *Oroxylum indicum* (L.) leaf extract in albino Wistar rats

**DOI:** 10.1186/s12906-022-03508-1

**Published:** 2022-04-09

**Authors:** Shalini Mohan, Kalaivani Thiagarajan, Balaji Sundaramoorthy, Vivek Gurung, Manas Barpande, Shilpi Agrawal, Rajasekaran Chandrasekaran

**Affiliations:** Department of Plant Biotechnology, School of Bio Sciences and Technology, VIT University, Vellore, Tamil Nadu 632014 India

**Correction to: BMC Complement Altern Med 16, 229 (2016)**

**https://doi.org/10.1186/s12906-016-1186-x**

Following publication of the original article [1], the authors identified an error in the author name of Shilpi Agrawal.

The incorrect author name is: Shilpi Agarwal

The correct author name is: Shilpi Agrawal

The original article has been corrected.
